# Estimated Costs for Delivery of HIV Antiretroviral Therapy to Individuals with CD4+ T-Cell Counts >350 cells/uL in Rural Uganda

**DOI:** 10.1371/journal.pone.0143433

**Published:** 2015-12-03

**Authors:** Vivek Jain, Wei Chang, Dathan M. Byonanebye, Asiphas Owaraganise, Ellon Twinomuhwezi, Gideon Amanyire, Douglas Black, Elliot Marseille, Moses R. Kamya, Diane V. Havlir, James G. Kahn

**Affiliations:** 1 HIV/AIDS Division, San Francisco General Hospital, University of California San Francisco (UCSF), San Francisco, CA, United States of America; 2 Makerere University-UCSF (MU-UCSF) Research Collaboration, Kampala, Uganda; 3 Philip R. Lee Institute for Health Policy Studies, UCSF, San Francisco, CA, United States of America; 4 Makerere University Joint AIDS Program (MJAP), Kampala, Uganda; 5 Health Strategies International, Oakland, CA, United States of America; 6 Department of Medicine, Makerere University College of Health Sciences, Kampala, Uganda; British Columbia Centre for Excellence in HIV/AIDS, CANADA

## Abstract

**Background:**

Evidence favoring earlier HIV ART initiation at high CD4+ T-cell counts (CD4>350/uL) has grown, and guidelines now recommend earlier HIV treatment. However, the cost of providing ART to individuals with CD4>350 in Sub-Saharan Africa has not been well estimated. This remains a major barrier to optimal global cost projections for accelerating the scale-up of ART. Our objective was to compute costs of ART delivery to high CD4+count individuals in a typical rural Ugandan health center-based HIV clinic, and use these data to construct scenarios of efficient ART scale-up.

**Methods:**

Within a clinical study evaluating streamlined ART delivery to 197 individuals with CD4+ cell counts >350 cells/uL (EARLI Study: NCT01479634) in Mbarara, Uganda, we performed a micro-costing analysis of administrative records, ART prices, and time-and-motion analysis of staff work patterns. We computed observed per-person-per-year (ppy) costs, and constructed models estimating costs under several increasingly efficient ART scale-up scenarios using local salaries, lowest drug prices, optimized patient loads, and inclusion of viral load (VL) testing.

**Findings:**

Among 197 individuals enrolled in the EARLI Study, median pre-ART CD4+ cell count was 569/uL (IQR 451–716). Observed ART delivery cost was $628 ppy at steady state. Models using local salaries and only core laboratory tests estimated costs of $529/$445 ppy (+/-VL testing, respectively). Models with lower salaries, lowest ART prices, and optimized healthcare worker schedules reduced costs by $100–200 ppy. Costs in a maximally efficient scale-up model were $320/$236 ppy (+/- VL testing). This included $39 for personnel, $106 for ART, $130/$46 for laboratory tests, and $46 for administrative/other costs. A key limitation of this study is its derivation and extrapolation of costs from one large rural treatment program of high CD4+ count individuals.

**Conclusions:**

In a Ugandan HIV clinic, ART delivery costs—including VL testing—for individuals with CD4>350 were similar to estimates from high-efficiency programs. In higher efficiency scale-up models, costs were substantially lower. These favorable costs may be achieved because high CD4+ count patients are often asymptomatic, facilitating more efficient streamlined ART delivery. Our work provides a framework for calculating costs of efficient ART scale-up models using accessible data from specific programs and regions.

## Introduction

The global scale-up of HIV antiretroviral therapy (ART) is continuing, with an estimated 13 million individuals now receiving medications [1, 2]. In 2013, WHO guidelines recommended expanding ART eligibility to all HIV-positive persons with CD4+ counts <500 and persons with CD4>500 who have tuberculosis or hepatitis B, pregnant or breastfeeding women, and persons in serodiscordant partnerships. [3] In 2015, WHO guidelines expanded further, recommending universal ART for all HIV-positive individuals regardless of disease stage, CD4+ count, or epidemiologic features [4].

This rapid global expansion in ART eligibility to higher CD4+ count individuals has generated robust discussion about projected ART delivery costs for the remaining 12–13 million infected individuals not yet on therapy in Sub-Saharan Africa, many of whom have higher CD4+ cell counts [5–9]. Data on this high CD4+ count population currently is lacking, as these individuals have only recently begun initiating ART. However, such data are crucially needed to guide modeling, forecasts, and the timing and level of future global ART expenditures.

Among lower CD4+ count individuals (CD4<350), ART delivery costs have been broadly estimated in low-income Sub-Saharan African countries. Estimates for median ART delivery costs have ranged from $646-$797 per-person per-year (ppy) in low income countries [10], and data from President’s Emergency Plan for AIDS Relief (PEPFAR)-supported clinics have shown median costs of $682-$988 ppy [11]. Other reports, however, have estimated lower costs of $200-$400 ppy [12–15]. These estimates, though heterogeneous, have informed modeling of the costs of ongoing ART scale-up.

Among high CD4+ count patients (CD4>350), a tacit assumption in cost estimations has been that annual ART costs will be similar to those for lower CD4+ count individuals [9]. However, this assumption may be incorrect for several reasons. The healthier clinical status of high CD4+ count patients at ART initiation may portend lower healthcare utilization costs because higher CD4+ patients (1) experience fewer clinical complications on ART, (2) achieve more robust immune recovery, (3) have lower hospitalization and healthcare utilization rates, and (4) have lower aggregate mortality [3, 16–19].

Even more fundamentally, the healthier clinical status of high CD4+ count patients could enable clinics to “streamline” care, i.e., deliver ART with less physician time, using nurse-driven models [20–23] and other interventions that promote efficiency, speed, and satisfaction while maintaining clinical efficacy. Several evidence-based streamlining interventions have emerged and are finding increasing adoption.

We report here results from a micro-costing study of ART delivery to asymptomatic high CD4+ count (>350) individuals in a prototypic rural HIV clinic in Uganda. To our knowledge, these are the first data informing ART costs specifically in high CD4+ count (>350) individuals. This population is expected to grow rapidly as guidelines continue to evolve, and as ART eligibility increases. Economic data on high CD4+ count individuals will increasingly inform and improve overall global ART cost calculations.

## Methods

### Study setting and intervention

The EARLI Study (www.clinicaltrials.gov NCT#01479634) [24] assessed ART efficacy and safety among individuals with CD4>350 starting in November, 2011. It is located at Bwizibwera Health Center in Mbarara, southwestern Uganda, a facility supported by the Makerere University Joint AIDS Program (MJAP), a PEPFAR implementing partner. EARLI Study inclusion criteria were previously reported [24], and included: (1) age ≥18 years, (2) asymptomatic clinical status, (3) residence <30km from the health center, (4) CD4≥350 cells/uL. Exclusion criteria included: (1) WHO stage 3/4 illness, and (2) pregnancy at study screening. EARLI was approved by ethical review boards of Makerere University, Kampala, Uganda and the University of California, San Francisco. All participants provided informed written consent for study participation.

Overall, 246 individuals enrolled in EARLI; 197 had CD4 ≥350 [24]. Patients with CD4≥350 initiated ART with Truvada (tenofovir/emtricitabine) with efavirenz, with optional Aluvia (lopinavir/ritonavir) if pregnancy, side effects, or virologic failure occurred. Participants with CD4<350 initiated standard Ugandan first-line ART (TDF/3TC/EFV or AZT/3TC/EFV).

EARLI Study participants were seen by a physician at ART initiation and at 4 weeks. A nurse then saw participants at weeks 8 and 12, and every 3 months for 3 years. As previously reported, nurse visits consisted of rapid focused questions that aimed to evaluate for ART side effects or medical complaints [24]. When these were absent, the visit was concluded and patient proceeded to a phlebotomist for blood draw and/or the pharmacist for ART refills. When ART side effects or medical problems were noted, the nurse could consider converting the visit to a physician evaluation. Safety laboratory evaluations were done along with HIV-1 plasma RNA determination (viral load, VL) twice per year. All viral load results were shared with patients when available, and discussed using a structured counseling script that focused on encouragement and positive reinforcement when viral load was undetectable, and counseling and adherence support when viral load was detectable [24].

These streamlined care procedures differed from standard practices in Western Uganda in two primary ways. First, patients received a tiered nurse-driven evaluation that had a built-in triggered physician backup, rather than all patients being seen by a composite team of nurses and physicians. Second, patients received viral load testing as well as structured counseling on viral load results, rather than no viral load testing.

### Micro-costing of ART delivery

We conducted a comprehensive accounting of resources consumed by ART-related activities, applying standard micro-costing methods that quantify each type of resource consumed (e.g., medications, lab tests and personnel time) and assign a unit cost [25]. Costing data were verified via interviews with study personnel, managers, and accountants.

#### Antiretroviral medication costs

Patients receiving first-line ART (tenofovir/emtricitabine/efavirenz) and second-line ART (tenofovir/emtricitabine/ritonavir/lopinavir) were totaled and multiplied by the ART regimen cost. Because tenofovir/emtricitabine was donated to the study, we substituted the cost of tenofovir/lamivudine reported in the Medicines Sans Frontiers (MSF) 2014 ‘Untangling the Web of Antiretroviral Price Reductions’ database [26]. Emtricitabine and lamivudine are pharmacologically equivalent and interchangeable medications [27]. We did not adjust costs for potential wastage; however, ART adherence was very high [24].

To estimate costs under various ART scale-up models (described below), we utilized the lowest available prices of first-line therapy (MSF 2014 database).

#### Laboratory monitoring costs

Laboratory test frequencies were derived from the EARLI Study protocol [28] and costs derived from administrative records. These reflect real resource consumption including reagent costs, expendable supplies, equipment depreciation, and incremental labor for each test.

Within our modeled ART scale-up scenarios, we used a standard twice-per-year complete blood count ($4.50) and CD4+ count ($8.50). For every model, we computed costs with and without a twice-per-year viral load test ($42 each).

#### Direct services personnel costs

Salary records were obtained from administrative records of the Infectious Diseases Research Collaboration (IDRC), a Ugandan non-profit organization providing logistic/administrative support to the study via the Makerere University-UCSF Research Collaboration. EARLI Study staff were salaried by IDRC. We quantified expenditures for support personnel, including both administrative and direct services support. For amortization, we assumed a personnel turnover rate of three years.

To estimate the personnel portion of total ART costs under modeled scale-up scenarios, we first utilized salary scales from the Makerere University Joint AIDS Program (MJAP). These are lower than EARLI Study salaries, and are more representative of PEPFAR-supported program activities. In other modeled scenarios, we utilized lowest possible salaries derived from current Uganda Ministry of Health salary scales. These are more broadly representative of health center worker salaries.

#### Administrative and other costs

Facility and infrastructural costs including rent, electricity, and water, were prorated by the fraction of the clinic square area used by the EARLI Study. Costs of office supplies, motorcycle maintenance and fuel, and ART storage at a central facility were also included. Expenditures not directly related to ART delivery were also evaluated, including salaries for central/administrative IDRC employees managing payroll, database support, and vehicle maintenance. Central costs were prorated to the fraction of staff equivalents spent supporting the EARLI Study.

Costs were converted from Ugandan shillings to US dollars at the June 30 exchange rate of the relevant year [29] and adjusted to 2012 US dollars via the consumer price index [30].

### Time and motion study

To model the potential gains in efficiency and resultant decreases in ART delivery costs that could be achieved if staff time was used at optimal efficiency, we conducted a time and motion study to analyze how staff members spent typical workdays, and to quantify how much time was spent idle that could theoretically be reassigned to patient care in modeled scenarios.

To compute the fraction of staff effort devoted to ART delivery, we designed a form with a comprehensive list of staff time expenditures. This was based on previously published instruments [31, 32]. Study staff were interviewed individually to enumerate work activities, which were coded alphanumerically. Staff ensured codes were mutually exclusive and exhaustive. Staff underwent training on using the form, and after a one-week pilot test, the form was refined further based on staff feedback. The activity list (30 individual codes), is given in S1 Appendix A: Time and Motion Study Activity Codes. Staff comprehensively recorded daily activities during a one-week period in 2012, and again during a 3-week period in 2014 (total 4 weeks observation).

Using time and motion data, we computed the fractional effort of each staff member’s day spent on ART delivery. In our modeled ART scale-up scenarios, we adjusted the per-person per-year cost of ART delivery by computing the percentage increase in available worker time per day, and multiplying by study enrollment. Based on that higher number of participants served, we re-computed average ppy costs. Due to increased efficiency, estimated ART delivery costs declined, as staff more fully utilized each work day.

Because staff recorded work days lasting <8 hours (consistent with workforce data from East African ART clinics [32, 33]), we also sought to assess the potential impact of working a full 8-hour day. We adjusted costs by computing the fraction of an 8-hour day worked, dividing the number of patients served in each model by this fraction. This yielded a higher number of patients served annually; from this, we re-computed average ppy costs for ART delivery.

### Modeling of costs in ART scale-up scenarios

Using the cost inputs described above, and adjustments that capture progressively higher levels of efficiency, we modeled six theoretical ART scale-up scenarios designed to approximate costs of ART delivery at large-scale under key conditions.

### Model A (steady-state patient load, Makerere Joint AIDS Program [MJAP] salary scale and standard core laboratory monitoring schedule)

ART delivery costs were computed as the sum of annualized ART, laboratory, personnel, and central/overhead costs. However, lower salary scales used by the MJAP ART Program were utilized for the model instead of the higher salaries paid by the EARLI Study. In addition, laboratory monitoring costs only included a twice-per-year CD4+ count and complete blood count, as recommended in Uganda national guidelines for ART monitoring [34] (rather than the laboratory monitoring scheduled dictated by the EARLI Study protocol).

### Model B (increased efficiency due to lowest available ARV drug costs)

ART costs were computed as in model A, however, lowest available ARV drug costs were substituted for maximal efficiency [26].

### Model C (increased efficiency by use of Ministry of Health salary scales)

ART costs were computed as in model B, however lower salary scales in use at the Uganda Ministry of Health (2013) were substituted for MJAP program salary scales.

### Model D (increased efficiency due to full use of workday)

ART costs were computed as in model C, except for an upward adjustment in the annualized number of patients cared for due to the full use of a workday [i.e., by replacing inactive/waiting time during the workday with patient care time (while preserving routine morning and lunch break time)], and a resulting downward adjustment in annualized ART cost.

### Model E (increased efficiency due to full use of 8-hour workday)

ART costs in this model were computed as in model D, except that the upward adjustment in number of patients served modeled a fully-utilized 8 hour day (with standard break times) rather than the as-reported length of workday.

### Model F (full use of 8-hour workday; higher salary)

ART costs in this model were identical to model E, except that a higher MJAP-scale salary was modeled on the assumption that operationalizing a full 8-hour workday under current MOH salary scales may pose challenges.

### Sensitivity analyses

Under all six models, to account for variations in the above modeled efficiencies, we conducted sensitivity analyses that assumed that the number of increased patients served would be 75% of predicted and 50% of predicted, and for each scenario, recomputed ART costs.

## Results

### Study demographics and clinical results

Overall, 197 individuals with CD4>350 enrolled in the EARLI Study from 2011–2012. As previously reported [24], the median age was 35 years (IQR, 29–41), 65% of participants were female, and 70% worked in agriculture. The median CD4+ count at ART initiation was 564 (IQR, 451–716). Overall, 194 participants initiated ART with tenofovir/emtricitabine + efavirenz, while 3 participants initiated ART with tenofovir/emtricitabine + ritonavir/lopinavir. During year 1 of the study, 16 individuals switched from first-line efavirenz to second line ritonavir/lopinavir. Self-reported adherence was 98% by 3-day pill recall. An undetectable viral load was achieved by 189/197 (95.9%) of participants at Week 24 following ART initiation, and in 189/195 (96.9%) of patients by Week 48 [24]. During Year 1, two deaths and eight hospitalizations occurred among 197 participants. Adverse events and ART toxicities were rare [24].

### Base case costs

Unit cost estimates for components of ART delivery are given in Table 1. The average cost of ART delivery as observed in the EARLI study during Year 1 was $987 per-person per-year (ppy). Excluding the $84 cost of viral load testing ($42/test, 2 tests per year), the ppy cost of ART was $903. The Year 1 time period included EARLI Study initiation, with full staffing throughout but a patient load that rose to full enrollment over six months. It also included several one-time costs of ART initiation (e.g., baseline laboratory tests not repeated subsequently). The average “steady state” cost of ART delivery (i.e., after full enrollment was achieved) was $628 ppy ($544 without VL testing).

**Table 1:** Unit Cost Estimates for Antiretroviral Therapy Delivery (2012 $US).

**Direct Services Personnel ($ per month)**			
	**EARLI study**	**MJAP**	**MOH**
Physician	$2,455.00	$1,083.00	$1,000.00
Nurse	$784.00	$602.00	$240.80
Pharmacy technician	$674.00	$602.00	$240.80
Laboratory technician	$674.00	$602.00	$240.80
Driver	$473.00	$277.00	$277.00
Home visitor	$487.00	$602.00	$240.80
**ARV Medications ($ per patient-month**)			
	**EARLI study**	**MSF best price**	
Truvada (TDF 300mg/FTC 200mg)	$5.74^A^	$5.74	
Efavirenz (EFV) 600mg	$7.20	$3.07	
Aluvia (LPV/RTV 200mg/50mg)	$49.00	$19.64	
**Lab Tests ($ per test)**			
Complete blood count	$4.50		
Electrolyte tests	$16.50		
Creatinine test	$9.00		
Liver function tests	$22.50		
Plasma HIV-1 RNA level	$42.00		
CD4+/CD8+ T cell counts	$8.50		
TB test (Xpert)	$20.40		
Urine pregnancy test	$3.00		
**Administration & Overhead ($ per month)**			
Offsite administration	$479.00		
General supplies	$351.00		
Motorcycle costs (incl. maintenance and fuel)	$169.00		
Equipment (microscope, centrifuge, glucometer, refrigerator, AC, biosafety cabinet)	$16.00		
Utilities	$130.00		
Rent (incl. security)	$160.00		
Storage for drugs at central office	$25.00		

**NOTE.** EARLI, ‘Early Antiretroviral Therapy in Resource Limited Settings in Patients with High CD4+ Counts’ Study; MJAP, Makerere Joint AIDS Program; MOH, Ministry of Health; TDF, tenofovir disoproxyl fumarate; FTC, emtricitabine; EFV, efavirenz; LPV, lopinavir; RTV, ritonavir; TB, tuberculosis; AC, airconditioning.

^A^: Truvada donated to the research study; MSF lowest price substituted.

### ART scale-up cost models (A-F)

#### Model A

**Fig 1:**
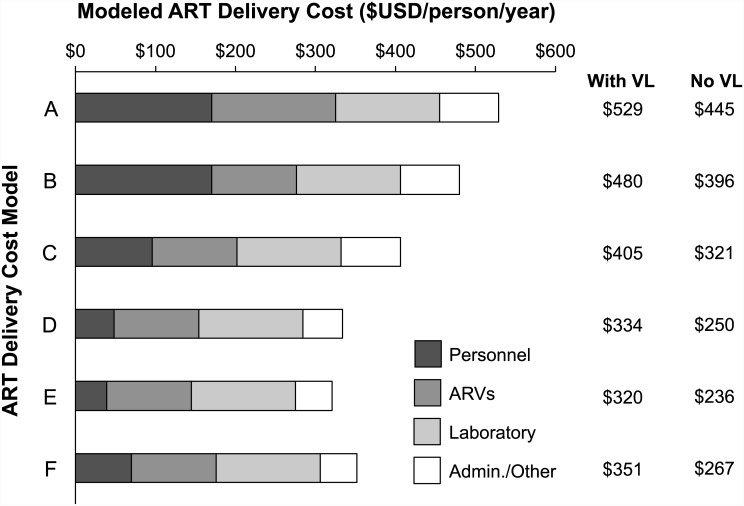
Estimated ART Delivery Costs under Modeled Scenarios of Efficient ART Scale-Up. Costs for ART delivery under six modeled scenarios are displayed inclusive of viral load testing. Model costs excluding viral load testing are also shown. **Model A:** steady-state patient load, MJAP program salary scale and standard core laboratory monitoring schedule. **Model B:** model A + lowest available ARV drug costs. **Model C:** model B + Uganda Ministry of Health 2013 salary scales. **Model D:** model C + increased healthcare worker efficiency due to full use of workday. **Model E:** model D + expansion of healthcare worker effort to full 8-hour workday. **Model F:** model E + higher MJAP program salary scales.

(increased efficiency due to use of a standard core laboratory monitoring schedule and MJAP salaries, with steady-state patient load) yielded a cost of $529 per-person per-year (Fig 1) [$445 ppy without VL measurements].

#### Model B

(Model A + lowest available ART costs) yielded a cost of $480 per-person per-year [$396 ppy without VL].

#### Model C

(Model B + increased efficiency using ministry of health salary scales) yielded a cost of $405 per-person per-year [$321 ppy without viral load measurements].

#### Model D

(Model C + increased efficiency due to full use of the available work day) yielded a projected increase in the number of patients served per year from 246 to 488. This resulted in a modeled cost of $334 per-person per-year [$250 ppy without VL].

#### Model E

(Model D + increased efficiency due to full use of an 8-hour work day) yielded a projected increase in the number of patients served per year from 488 to 602. This yielded a cost of $320 per-person per-year [$236 ppy without VL].

#### Model F

(Model E + lower efficiency due to use of higher MJAP salary scales) yielded a cost of $351 [$267 ppy without VL]. Sensitivity analyses assessing 75% scale-up efficiency and 50% scale-up efficiency scenarios showed slightly higher overall ART costs (Table 2).

**Table 2:** Sensitivity Analyses of Varying Efficiency/Gain Scenarios.

		*100% Efficiency*		*75% Efficiency*		*50% Efficiency*	
Modeled Scenario		With VL	No VL	With VL	No VL	With VL	No VL
**A:**	Observed cost with MJAP salary scales & standard lab monitoring	$529	$445	—	—	—	—
**B:**	Scenario A with lowest ARV prices	$480	$396	—	—	—	—
**C:**	Scenario B with MOH salary scales	$405	$321	—	—	—	—
**D:**	Scenario C with increased efficiency due to full use of workday	$334	$250	$344	$260	$358	$274
**E:**	Scenario D with increased efficiency due to full use of 8-hour workday	$320	$236	$331	$247	$345	$261
**F:**	Scenario E with MJAP salary level	$351	$267	$366	$282	$388	$304

**NOTE.** VL, viral load (HIV RNA level); MJAP Makerere Joint AIDS Program; ARV, antiretroviral (drug); MOH, ministry of health.

## Discussion

Although antiretroviral therapy (ART) access is expanding to higher CD4+ count HIV-positive patients (CD4>350), data on the costs associated with treating this population have been lacking, limiting calculations of the financial resources needed to meet the demands of the ongoing ART scale-up in Sub-Saharan Africa.

We report here ART micro-costing results from a study of patients with CD4>350 receiving ART in a prototypic Ugandan HIV clinic via a nurse-driven streamlined system of ART delivery that achieved 95% virologic suppression at one year [24]. Using programmatic cost data to generate models that examine increasingly efficient ART delivery scenarios that could occur at wide scale, we project steady-state annual ART delivery costs of $320-$530/person/year. These estimates for high CD4+ count patients are similar to or lower than published estimates for lower CD4+ count patients, despite augmentations of care including twice-per-year viral load measurement, a telephone hotline for patients to contact the clinic with questions, and motorcycle-based tracking of patients who miss clinic visits. To our knowledge, these data are among the first to describe ART delivery costs for individuals with CD4>350, and have the potential to inform modeling of ART costs as the treatment scale-up continues.

HIV-positive individuals with CD4+ counts >350 experience fewer complications with ART initiation and subsequent therapy than lower CD4+ count patients [16], and have better immunologic recovery [35, 36]. They have excellent adherence, and are retained in care at substantial rates [24, 37–39]. As such, they are distinctly amenable to interventions that streamline HIV care. Several such interventions have proven efficacious and are now being expanded including nurse-driven systems [20, 22, 23, 40] that use less intensive laboratory monitoring, and allow longer refill durations and fewer visits per year [39].

Streamlining innovations can increase cost efficiency by allowing treatment of greater numbers of patients with fewer net resources. As ART eligibility expands, streamlined systems will likely play an increasingly important role in the ART landscape. Efforts to increase diagnosis of HIV-positive individuals, for example via large-scale community testing campaigns, will also play a crucial role The modeled ART cost scenarios in our report offer the ability to test the cost implications of different streamlining strategies, and adjust these as costs of all components of care improve over time. Our modeled scenarios reveal several notable findings.

Dramatic reductions have occurred in the prices of ART medications, fueled by progress in generic manufacturing and importation [26]. Despite this progress, ART medication cost remains substantial, comprising 18–33% in our modeled scenarios. Further reductions in ART costs are expected to occur, as generic manufacturing advances, and particularly as fewer pregnant women shift from inexpensive first-line therapy (e.g., with efavirenz) to second-line therapy (generally with protease inhibitors). Our model-based approach may allow for rapid adjustment of predicted costs using latest pricing information.

Personnel costs constitute a substantial fraction of total ART delivery costs in our models. As the shift from model B to model C shows (shifting to ministry of health level salaries), however, large efficiencies can arise by using lower-salary clinicians. Streamlined models of ART care may be an optimal way to maximize patient load per unit of personnel cost; i.e., by drawing upon nurse-driven systems with tiered physician support—either in person or via telephone hotlines [41], allowing higher-salary clinicians to serve more centrally, thus reaching a greater number of patients.

Full utilization of budgeted personnel time is also a crucial aspect to maximizing efficiency, as illustrated by the decrease in costs moving from model C to model D (utilizing the full work day). Better matching of patient load to available personnel can achieve this; however, with increasing efforts to decentralize care, this becomes logistically more difficult. Fostering the optimal patient-nurse-physician ratio is challenging even at centralized facilities; it is even more so at more peripheral facilities that are being prioritized for expansion in many regions.

A different but related consideration in ART delivery costs is the time considered to represent a full clinic work day. As our data show, shifting from model D to E (moving to an appointed 8-hour work day) created further cost savings. However, prior studies have documented persistent challenges for clinicians completing a full-time work week [42]. Competing demands of insufficient salaries, infrastructural challenges, and training and feedback gaps may drive clinicians to pursue alternative secondary employment or other activities that shorten the workday [42]. To the extent that this type of workforce shortage can be addressed by increasing salaries and working conditions, our modeling approach may help capture this interplay.

Laboratory related costs also continue to be major drivers of overall cost. Currently, chief among these is HIV-1 plasma RNA quantitation (viral load, VL), which is now recommended for universal adoption into HIV care [3, 43] but whose expense remains high. Innovative new approaches to VL testing—including many novel point of care approaches [44]—promise to substantially reduce VL costs. But as adoption increases, modeling approaches will be needed to reassess costs derived from VL testing. In our view, VL testing, while currently expensive, was a crucial element that allowed streamlining of care in other domains. For example, repeated demonstration of undetectable VL in a patient can allow for rapid nurse-driven visits, less intense adherence counseling, and faster transit time through clinic, all of which increase cost efficiency. In addition, our modeling included twice-per-year CD4+ count measurement. Recent research has demonstrated the questionable benefit of CD4+ monitoring once viral suppression is achieved [45, 46], and WHO has advised reducing or eliminating CD4+ monitoring in stable patients [43]. Thus, both from the standpoint of decreasing viral load costs, and from the standpoint of reducing CD4+ monitoring, our cost estimates are likely to be conservative.

Our study had certain limitations. First, we did not compute costs for participant hospitalizations; however, these were rare, occurring in only 8/197 participants through Year 1 [24], consistent with recently published data on low clinical event rates in patients treated at higher CD4+ counts [16]. Second, our data are derived from one clinic. While this clinic represents a prototypic government-run facility serving a rural East African population, broader data on high CD4+ count patients are needed, particularly from more urbanized regions. These data may soon emerge from programs treating asymptomatic high CD4+ count individuals who are members of sero-discordant couples, as this is a key population being prioritized for high CD4+ treatment. Third, our study was not designed to capture patient-centric economic costs of attending clinic (e.g., transportation costs), costs of testing and recruiting new patients (e.g. via expanded community testing initiatives), or cost-saving benefits of streamlined care (e.g., lower opportunity cost due to missing fewer hours of work). Finally, the high adherence seen in our clinic population may not represent all clinic settings. Lower adherence could blunt some of the efficiency gains we modeled. However, adherence and viral suppression rates of 85–90% and higher are increasingly being demonstrated in Uganda.[47–49] Future cost estimations may take these secondary factors into account—particularly as they relate to asymptomatic high CD4+ count individuals.

In summary we provide the first data to our knowledge on estimated costs of ART delivery to high CD4+ count individuals (CD4>350) in a prototypic rural East African clinic setting. We find costs that include viral load monitoring to be similar to diverse estimates from efficient programs that do not include viral load. We also model costs of optimized large-scale ART delivery with maximal staff efficiency and patient loads, and find these costs to be within the lower range of published ART costs.

These data should encourage continued ART expansion into asymptomatic high CD4+ count populations, and should inform updated calculations of the global financial resources needed to meet the coming demands of reaching universal ART coverage throughout Sub-Saharan Africa.

## Supporting Information

S1 Appendix A Time and motion study activity codes. (PDF)

S1 Dataset Master dataset underlying microcosting calculations. (PDF)
