# Supplementary material for: Estimated Costs for Delivery of HIV Antiretroviral Therapy to Individuals with CD4+ T-Cell Counts >350 cells/uL in Rural Uganda
Source: PLoS One. 2015 Dec 3;10(12):e0143433. doi: 10.1371/journal.pone.0143433 (PMC4669141; doi:10.1371/journal.pone.0143433)
Supplement: S1 Appendix A — (PDF) [file pone.0143433.s001.pdf]

S1 Appendix A: Time and Motion Study Activity Codes

Code	Activity
PCA	Patient (Pt.) consultation: ART initiation
PCR	Pt. consultation: ART management
PCS	Pt. consultation: ART side effects
PCD	Pt. consultation: non-ART
MR	Medical record data entry
TC	Telephone: clinical
TL	Telephone: logistics
TT	Telephone: tracking
ECR	Email: clinical
MDA	Medication: ART dispensing
MDE	Medication: non-ART dispensing
MSR	Medication: stocking/recordkeeping
MPP	Medication: pre-packing
PGT	Pt. greeting/triage
LD	Lab: phlebotomy
LSP	Lab: specimen processing
LG	Lab: general set-up/maintenance
LP	Lab: paperwork
LRR	Lab: results review
LN	Lab: non EARLI study
TRS	Transport: samples
TRL	Transport: logistics
HV	Tracking: home visit
RPR	Review patient records
MG	Staff meeting
FG	Filing
GO	General office work
RW	Regulatory work
WT	Waiting time between patients/tasks
BR	Break
