# Supplementary material for: Estimated Costs for Delivery of HIV Antiretroviral Therapy to Individuals with CD4+ T-Cell Counts >350 cells/uL in Rural Uganda
Source: PLoS One. 2015 Dec 3;10(12):e0143433. doi: 10.1371/journal.pone.0143433 (PMC4669141; doi:10.1371/journal.pone.0143433)
Supplement: S1 Dataset — (PDF) [file pone.0143433.s002.pdf]

S1 Dataset. Master dataset underlying microcosting calculations.

Jain, V. et al; *Estimated Costs for Delivery of HIV Antiretroviral Therapy to Individuals with CD4+ T-Cell Counts >350 cells/uL in Rural Uganda*; PLOS ONE

	Observed EARLI ART Delivery Cost				Steady-State EARLI ART Delivery Cost			
Item	Units	Unit cost	Monthly cost	Yearly cost per patient	Units	Unit cost	Monthly cost	Yearly cost per patient
Direct Services Personnel	***FTE***	***USD/mo.***		***$377***	***FTE***	***USD/mo.***		***$248***
Doctor	1.00	$2,455	$2,455	$182	1.00	$2,455	$2,455	$120
Nurse	1.00	$784	$784	$58	1.00	$784	$784	$38
Pharm technician	1.00	$674	$674	$50	1.00	$674	$674	$33
Lab technician	1.00	$674	$674	$50	1.00	$674	$674	$33
Home visitor	1.00	$487	$487	$36	1.00	$487	$487	$24
ARV Medications	***# pt.-mo.***	***USD/mo.***		***$180.36***	***# pt.-mo.***	***USD/mo.***		***$180.36***
Efavirenz + Truvada	154	12.94	1,987	$147.52	234	12.94	3,024	$147.52
Aluvia + Truvada	8	54.74	442	$32.84	12	54.74	673	$32.84
Laboratory Tests	***# pt.-mo.***	***USD/mo.***		***$331***	***# pt.-mo.***	***USD/mo.***		***$135***
Lab-Male	58	$25	$1,482	$110	89	$10	$871	$42
Lab- Female	103	$26	$2,713	$201	157	$10	$1,627	$79
Lab supplies	1.00	$268	$268	$20	1.00	$268	$268	$13
Administrative/Other Costs		***USD/mo.***		***$99***		***USD/mo.***		***$65***
Offsite admin	1.00	$479	$479	$36	1.00	$479	$479	$23
General supplies	1.00	$351	$351	$26	1.00	$351	$351	$17
Vehicle - Maintenance	1	$30	$30	$2	1	$30	$30	$1
Vehicle - Fuel	1	$125	$125	$9	1	$125	$125	$6
Utilities	1	$130	$130	$10	1	$130	$130	$6
Rent - Clinic (incl. security)	1	$160	$160	$12	1	$160	$160	$8
Storage at MJAP	1	$25	$25	$2	1	$25	$25	$1
Vehicle	1	$14	$14	$1	1	$14	$14	$1
Equipment	1	$16	$16	$1	1	$16	$16	$1
**TOTAL COST PPY**				**$987.08**				**$627.74**

	Scenario A: Observed cost with MJAP salary scales & standard laboratory monitoring				Scenario B: Scenario A with lowest ARV prices			
Item	Units	Unit cost	Monthly cost	Yearly cost per patient	Units	Unit cost	Monthly cost	Yearly cost per patient
Direct Services Personnel	***FTE***	***USD/mo.***		***$170***	***FTE***	***USD/mo.***		***$170***
Doctor	1.00	$1,083	$1,083	$53	1.00	$1,083	$1,083	$53
Nurse	1.00	$602	$602	$29	1.00	$602	$602	$29
Pharm technician	1.00	$602	$602	$29	1.00	$602	$602	$29
Lab technician	1.00	$602	$602	$29	1.00	$602	$602	$29
Home visitor	1.00	$602	$602	$29	1.00	$602	$602	$29
ARV Medications	***# pt.-mo.***	***USD/mo.***		***$155***	***# pt.-mo.***	***USD/mo.***		***$106***
Efavirenz + Truvada	246	$13	$3,183	$155	246	$9	$2,167	$106
Aluvia + Truvada	0	$55	$0	$0	0	$25	$0	$0
Laboratory Tests	***# pt.-mo.***	***USD/mo.***		***$130***	***# pt.-mo.***	***USD/mo.***		***$130***
Lab-Male	89	$9	$816	$40	89	$9	$816	$40
Lab- Female	157	$9	$1,439	$70	157	$9	$1,439	$70
Lab supplies	1.52	$268	$408	$20	1.52	$268	$408	$20
Administrative/Other Costs		***USD/mo.***		***$74***		***USD/mo.***		***$74***
Offsite admin	1.00	$479	$479	$23	1.00	$479	$479	$23
General supplies	1.52	$351	$535	$26	1.52	$351	$535	$26
Vehicle - Maintenance	1	$30	$30	$1	1	$30	$30	$1
Vehicle - Fuel	1	$125	$125	$6	1	$125	$125	$6
Utilities	1	$130	$130	$6	1	$130	$130	$6
Rent - Clinic (incl. security)	1	$160	$160	$8	1	$160	$160	$8
Storage at MJAP	1	$25	$25	$1	1	$25	$25	$1
Vehicle	1	$14	$14	$1	1	$14	$14	$1
Equipment	1	$16	$16	$1	1	$16	$16	$1
**TOTAL COST PPY**				**$529.37**				**$479.81**

	Scenario C: Scenario B with MOH salary scales				Scenario D: Scenario C with increased efficiency due to full use of workday			
Item	Units	Unit cost	Monthly cost	Yearly cost per patient	Units	Unit cost	Monthly cost	Yearly cost per patient
Direct Services Personnel	***FTE***	***USD/mo.***		***$96***	***FTE***	***USD/mo.***		***$48***
Doctor	1.00	$1,000	$1,000	$49	1.00	$1,000	$1,000	$25
Nurse	1.00	$241	$241	$12	1.00	$241	$241	$6
Pharm technician	1.00	$241	$241	$12	1.00	$241	$241	$6
Lab technician	1.00	$241	$241	$12	1.00	$241	$241	$6
Home visitor	1.00	$241	$241	$12	1.00	$241	$241	$6
ARV Medications	***# pt.-mo.***	***USD/mo.***		***$106***	***# pt.-mo.***	***USD/mo.***		***$106***
Efavirenz + Truvada	246	$9	$2,167	$106	488	$9	$4,299	$106
Aluvia + Truvada	0	$25	$0	$0	0	$25	$0	$0
Laboratory Tests	***# pt.-mo.***	***USD/mo.***		***$130***	***# pt.-mo.***	***USD/mo.***		***$130***
Lab-Male	89	$9	$816	$40	176	$9	$1,613	$40
Lab- Female	157	$9	$1,439	$70	312	$9	$2,860	$70
Lab supplies	1.52	$268	$408	$20	3.02	$268	$810	$20
Administrative/Other Costs		***USD/mo.***		***$74***		***USD/mo.***		***$50***
Offsite admin	1.00	$479	$479	$23	1.00	$479	$479	$12
General supplies	1.52	$351	$535	$26	3.02	$351	$1,061	$26
Vehicle - Maintenance	1	$30	$30	$1	1	$30	$30	$1
Vehicle - Fuel	1	$125	$125	$6	1	$125	$125	$3
Utilities	1	$130	$130	$6	1	$130	$130	$3
Rent - Clinic (incl. security)	1	$160	$160	$8	1	$160	$160	$4
Storage at MJAP	1	$25	$25	$1	1	$25	$25	$1
Vehicle	1	$14	$14	$1	1	$14	$14	$0
Equipment	1	$16	$16	$1	1	$16	$16	$0
**TOTAL COST PPY**				**$405.28**				**$334.09**

	Scenario E: Scenario D with increased efficiency due to full use of 8-hour workday				Scenario F: Scenario E with MJAP salary level			
Item	Units	Unit cost	Monthly cost	Yearly cost per patient	Units	Unit cost	Monthly cost	Yearly cost per patient
Direct Services Personnel	***FTE***	***USD/mo.***		***$39***	***FTE***	***USD/mo.***		***$70***
Doctor	1.00	$1,000	$1,000	$20	1.00	$1,083	$1,083	$22
Nurse	1.00	$241	$241	$5	1.00	$602	$602	$12
Pharm technician	1.00	$241	$241	$5	1.00	$602	$602	$12
Lab technician	1.00	$241	$241	$5	1.00	$602	$602	$12
Home visitor	1.00	$241	$241	$5	1.00	$602	$602	$12
ARV Medications	***# pt.-mo.***	***USD/mo.***		***$106***	***# pt.-mo.***	***USD/mo.***		***$106***
Efavirenz + Truvada	602	$9	$5,304	$106	602	$9	$5,304	$106
Aluvia + Truvada	0	$25	$0	$0	0	$25	$0	$0
Laboratory Tests	***# pt.-mo.***	***USD/mo.***		***$130***	***# pt.-mo.***	***USD/mo.***		***$130***
Lab-Male	217	$9	$1,989	$40	217	$9	$1,989	$40
Lab- Female	385	$9	$3,529	$70	385	$9	$3,529	$70
Lab supplies	3.72	$268	$999	$20	3.72	$268	$999	$20
Administrative/Other Costs		***USD/mo.***		***$46***		***USD/mo.***		***$46***
Offsite admin	1.00	$479	$479	$10	1.00	$479	$479	$10
General supplies	3.72	$351	$1,308	$26	3.72	$351	$1,308	$26
Vehicle - Maintenance	1	$30	$30	$1	1	$30	$30	$1
Vehicle - Fuel	1	$125	$125	$2	1	$125	$125	$2
Utilities	1	$130	$130	$3	1	$130	$130	$3
Rent - Clinic (incl. security)	1	$160	$160	$3	1	$160	$160	$3
Storage at MJAP	1	$25	$25	$0	1	$25	$25	$0
Vehicle	1	$14	$14	$0	1	$14	$14	$0
Equipment	1	$16	$16	$0	1	$16	$16	$0
**TOTAL COST PPY**				**$320.38**				**$350.84**

Abbreviations:
ART: antiretroviral therapy
ARV: antiretroviral (medication)
MJAP: Makerere Joint AIDS Program
MOH: Ministry of Health
FTE: full time equivalent
USD: United States dollar
Mo.: month
Pt.-mo.: patient-month
PPY: per person per year
